# Biomarkers of Deoxynivalenol, Citrinin, Ochratoxin A and Zearalenone in Pigs after Exposure to Naturally Contaminated Feed Close to Guidance Values

**DOI:** 10.3390/toxins13110750

**Published:** 2021-10-22

**Authors:** Agnieszka Tkaczyk, Piotr Jedziniak, Łukasz Zielonka, Michał Dąbrowski, Piotr Ochodzki, Adrianna Rudawska

**Affiliations:** 1 Department of Pharmacology and Toxicology, National Veterinary Research Institute, 24-100 Pulawy, Poland; piotr.jedziniak@piwet.pulawy.pl (P.J.); adrianna.rudawska@piwet.pulawy.pl (A.R.); 2 Department of Veterinary Prevention and Feed Hygiene, Faculty of Veterinary Medicine, University of Warmia and Mazury, 10-718 Olsztyn, Poland; lukaszz@uwm.edu.pl (Ł.Z.); michal.dabrowski@uwm.edu.pl (M.D.); 3 Department of Plant Pathology, Plant Breeding and Acclimatization Institute—National Research Institute, 05-870 Błonie, Poland; p.ochodzki@ihar.edu.pl

**Keywords:** mycotoxin biomarkers, pig’s exposure, multi-mycotoxin LC-MS/MS method, deoxynivalenol, zearalenone, ochratoxin A, citrinin

## Abstract

This study applied multi-mycotoxin liquid chromatography with tandem mass spectrometric detection (LC-MS/MS) methods to determine the biomarkers of exposure in urine and serum samples from a dose-response study with pigs. The 24 studied pigs were divided into three groups: a control and two experimental ones (with different levels of feed contamination). They were exposed to feed prepared from cereals contaminated with deoxynivalenol (DON), zearalenone (ZEN), ochratoxin A (OTA) and citrinin (CIT) for 14 days. After that, both experimental groups received the same feed as the control group for the next 14 days to determine the kinetics of the disappearance of mycotoxin biomarkers. Urine samples were collected daily in the morning and blood samples—eight-times during the experiment. The study reported herein was the first prolonged exposure experiment for multiple mycotoxins like OTA and CIT in pigs. The urinary and serum levels of all biomarkers correlated well with the respective toxin intake; thereby demonstrating that they are suitable biomarkers of exposure in pigs. Urine is a good candidate to monitor DON, ZEN, OTA, CIT exposure while serum may be used to monitor DON, OTA and CIT. Additionally, OTA has even been quantified in both matrices in the experimental groups two weeks after changing the contaminated feed back to the control, this result differed from those produced by the other mycotoxins which were only quantified during the first two weeks. Therefore both matrices are suitable candidates to monitor prolonged OTA exposure in pigs.

## 1. Introduction

Mycotoxins are commonly found in cereals, which are essential feed ingredients. Pigs are important animals for livestock production, susceptible to the harmful effects of mycotoxins [1]. Due to their cereal-based diet, they are exposed to these toxins and chronic exposure [2]. Mycotoxins have a wide range of adverse effects and may lead to significant economic losses in the agricultural sector [3]. Moreover, in toxicology studies, pigs serve as an important experimental model for humans due to their analogies in terms of gastrointestinal tract, liver and kidney functions [4,5].

According to recent studies, the majority of feed samples are co-contaminated with multiple mycotoxins [6]. Current legislation concerning animal feed only applies to compounds commonly known as “regulated toxins”: aflatoxins (AFLs) (maximum allowed level), deoxynivalenol (DON), T-2 toxin (T-2) and HT-2 toxin (HT-2), zearalenone (ZEN), fumonisins (FBs) and ochratoxin A (OTA) (guidance values) [7], because of their acute and chronic toxic effects [8].

The analysis of mycotoxin levels in the feed is commonly used to assess pig’ exposure to mycotoxins. This approach has some disadvantages, e.g., it does not include individual exposure or account for inhomogeneous distribution of a toxin in feedstuffs. Therefore an exposure assessment of animals may combine an analysis of toxin levels in the feed with an analysis of biological samples (blood, urine or faeces).

In recent times, many multi-mycotoxin methods designed to analyse biomarkers in pig biological matrices have been developed [9,10,11]. They were applied to study the following: the efficacy testing of the mycotoxin detoxifier [12], the metabolism of the modified mycotoxins [13,14,15] and pig biomonitoring [16].

Apart from these applications, it is also essential to provide the relationships between the toxin levels in the biological matrices and the toxin intake. These correalations are essential to calculate the toxin levels in diets using the biomarker levels quantified in biological matrices. This data could be used to establish future guidance values for mycotoxins in biological matrices.

In recent times, there have been four dose-response studies in pigs with four/five ZEN and DON feeding groups [17,18,19,20]. Additionally, only one experiment included exposure to OTA, fumonisin B_1_ (FB_1_) and aflatoxin B_1_ (AFB_1_) [21]. Mycotoxin biomarkers were analysed in urine in each of these studies and plasma in three studies [17,18,19]. High-performance liquid chromatography with tandem mass spectrometric detection (HPLC-MS/MS) analyses were carried out in every study with the exception of two HPLC methods [18,19].

In most cases, after a one-week acclimation period, for most of the studies, mycotoxins were administered mainly to small groups of pigs (four pigs per group) for 2.5–37 days (Supplementary Materials Table S1). However, two studies involved significantly more pigs—more than a hundred female piglets—divided into different feedings groups which resulted in more than 20 pigs per group [17,18]. The pigs were housed individually in metabolic cages [21] or pens [17,18]. The feed used in the experiments was prepared in such a way that it contained cereals, such as wheat [19] or maize [17,18,20] that were naturally contaminated with *Fusarium*. Although, in one of the studies, feed boluses fortified with mycotoxins were administered to pigs [21]. The maximum sampling time for all matrices lasted from exposure until 2 days (48 h) after ingestion of contaminated feed..

In contrast to other mycotoxins the DON and ZEN urinary and serum biomarkers have been frequently studied. Nevertheless, due to different experiment designs and often high degrees of variation within experimental groups, it is difficult to find DON and ZEN urinary and serum levels which correspond to their EU guidance values in feed (Supplementary Materials Table S1). This is of prime importance, because to date, no guidance values for the levels of these biomarkers in biological matrices have been established. The data concerning urinary biomarkers are not available for citrinin (CIT). Moreover, European Food Safety Authority (EFSA) published a scientific opinion article on CIT, which expressed the need for well-designed toxicological studies with laboratory animal species which further explores the toxicological potential of citrinin and characterizes the dose-response relationships [22]. In the recent scientific opinion article, the EFSA concluded that more studies describing biomarkers of exposure of OTA in blood to external daily intakes are required to reach decent assessment of OTA exposure [23].

The aim of our experiment with pigs was to:

(1) Find a reliable correlation between the concentration of mycotoxin in contaminated feed and mycotoxin biomarker levels in biological matrices (urine and serum) after the simultaneous oral ingestion of multiple mycotoxins: DON, ZEN (at concentrations close to the guidance values), OTA and CIT.
(2) Select adequate pig urinary and serum biomarkers for OTA and CIT.
(3) Check whether exposure time affected the toxin levels in the serum and urine of pigs (to establish adequate sampling time).
(4) Assess the kinetics of the disappearance of mycotoxin biomarkers in urine and serum samples for multiple mycotoxins (with the initial data being attained for urinary OTA and CIT biomarkers). In order to ensure conditions as close to the natural ones as possible, the experimental feed was prepared from cereals–corn—which was naturally contaminated with mycotoxins and rye–inoculated in the field with the *Fusarium* species.

## 2. Results and Discussion

### 2.1. Development of a Multi-Mycotoxin Method for the Quantification of Biomarkers in Pig Serum Samples

From several of sample preparation methods for mycotoxin quantification in pig serum, which have been reported recently in the literature, extraction with acetonitrile (ACN) [24,25,26,27,28] or acidified acetonitrile (0.1% formic acid (HCOOH)) [29] as the extraction solvent was the most popular method. The solid-phase extraction (SPE) columns were applied only once to determine DON, ZEN, and its metabolites after enzymatic hydrolysis [30].

The majority of mycotoxins occur in serum in a free form. In contrast to DON—the degree of conjugation of DON in serum found in the previous study was approximately 33% (19–45%) [19]. Nevertheless, enzymatic hydrolysis was only carried out once (with β-glucuronidase from *Helix pomatia*) in order to quantify ZEN and its metabolites [26]. Out of two multi-mycotoxin methods [25,29]—only one of them includes the direct determination of phase I and II metabolites by LC-high resolution mass spectrometry [29].

After the analysis of spiked serum and plasma samples at a medium QC level (Supplementary Materials Table S2) (extraction with ACN), the serum was selected for future investigation due to high signal enhancement for a majority of analytes and low extraction recoveries for aflatoxins (20–52%) from plasma samples compared to the serum (51–79%).

Then, different extraction solvents were tested in term of their extraction efficiency using urine samples (*n* = 3 in three replicates) spiked at a medium QC level (Supplementary Materials Table S2) before and after the extractions.

Similar recovery values were achieved with ACN, acidified ACN (0.1% HCOOH) (34–98%), methanol (MeOH) and ACN:MeOH (1:1) for most of the analytes studied. The addition of ethyl acetate (EtAc) resulted in an extremely low extraction recovery of OTA (1%). However, the recovery of aflatoxins slightly decreased from 51–70% (acidified ACN) to 41–60% when ACN was used. Therefore, acidified ACN (0.1% HCOOH) was used for the extraction—which was similar to the recent multi-mycotoxin LC-MS method used to determine 24 mycotoxins and their metabolites in pig serum [29].

Next, different enzymatic hydrolysis times and temperatures (37 °C, 18 h) after hydrolysis with acidified ACN (0.1% HCOOH) were compared with the initial conditions (60 °C, 3 h). Longer hydrolysis time resulted in lower R_E_ values for aflatoxin G_1_ (AFG_1_), aflatoxin G_2_ (AFG_2_) and OTA than the initial conditions applied in a later analysis.

The developed method was successfully validated (Supplementary Materials Tables S2 and S3).

### 2.2. Urinary and Serum Mycotoxin Biomarkers

As shown in Table 1, the concentration in the 24-h urine and serum samples of all biomarkers correlated well with the corresponding toxin intake, thereby demonstrating that they are suitable biomarkers of exposure in pigs. There were significant differences between the experimental groups and the control group (*p* < 0.05). Only traces of DON and ZEN were quantified in urine as well as OTA and CIT in serum obtained from the control group, which confirmed the suitability of the experiment design (Table 2 and Table 3).

All urine and serum samples were analysed after enzymatic hydrolysis, and the mycotoxin concentrations were expressed as the sum of the conjugated and unconjugated forms.

The creatinine concentrations were lower than 1 mg/mL in all analysed urine samples —with the mean concentration for: control group—0.34 ± 0.25 mg/mL, D1 group: 0.24 ± 0.15 mg/mL and D2 group: 0.28 ± 0.22 mg/mL—was much lower than the value recently described in pigs (0.07–10.77 mg/mL) [31]. The reason for that result could be that the 24-h urine sample was collected in this experiment in contrast to the recently analysed urine samples [31]. The relative standard deviations of urinary biomarker concentrations standardized to the creatinine level were high, thereby indicating that standardization is not applicable for 24-h urine samples.

Biomonitoring the level of mycotoxins in serum samples should include information from toxicokinetic studies in order to determine the period of time during which we can detect mycotoxins in serum (this applies to DON and ZEN, because of their rapid clearance from blood) or when the maximum concentration of toxins occurs (as for OTA and CIT, because of their long elimination half-life).

According to recent pharmacokinetic studies—the maximum level of DON in plasma was quantified after 3–4 h and decreased rapidly after that [12,14]. Therefore, in our experiment, blood was sampled about 3–4 h after the pig’s exposure to naturally contaminated feed.

#### 2.2.1. Deoxynivalenol Biomarkers

DON and deepoxy-deoxynivalenol (DOM-1) are the most important DON urinary biomarkers (Table 1). This assertion is in accordance with recent studies [17,18,19], however, the urinary DON and DOM-1 concentrations obtained in our study were much higher (Supplementary Materials Table S1) than in previously reported studies. This may be due to different experimental designs—a urine sample was only taken once—before slaughtering (3–4 h after feeding). Additionally, in two experiments, high-performance liquid chromatography with diode array detector (HPLC-DAD) methods [18,19] were used for biomarkers analysis. In one experiment, in which an LC-MS/MS method was applied, a high degree of variation was observed within the group [17] (Supplementary Materials Table S1). This is in contrast to our results—the relative standard deviation (RSD) values of DON and DOM-1 means of the urinary concentrations from the 14-day experimental period were lower than 25% for both experimental groups.

In another dose-response study, urinary concentrations of DON were about four times higher than in our study [21]. In our study, the naturally contaminated feed was used for the experiment in contrast to feed boluses fortified with pure cultures in the aforementioned study. This difference in experiment design could explain the higher DON urinary concentrations as compared to our results. However, the DOM-1 urinary concentrations were similar to our findings.

Pigs are not able to effectively degrade DON to DOM-1, therefore are particulary susceptible to DON [32]. Indeed, the proportion of DOM-1 in urine as a percentage of the sum of DON and DOM-1 level was 15.5%, and 20.4% for the D1 (lower level) and D2 (higher level) groups—these findings were comparable to those of a recent study (8.1-26.1%) [17].

DON is the only biomarker found in pig serum samples (Table 2). DOM-1 wasn’t detected in serum at dietary DON levels lower than the EU guidance value (900 µg DON/kg diet—D1 group), according to the results of other studies (Supplementary Materials Table S1). Dietary DON concentrations above the guidance value increased the maximum serum DOM-1 concentrations, whereby DOM-1 was only detected in 8.3%, 57% and 75% of the animals feeding with 1270, 2010 and 4520 µg DON per kg of their diet, respectively [33]. In our experiment with the D2 group, pigs ingested 1126 ± 91.43 µg DON/kg feed. Therefore DOM-1 was detected sporadically at concentrations below LOQ (0.8 ng/mL).

#### 2.2.2. Zearalenone Biomarkers

ZEN and its metabolite alpha-zearalenol (α-ZEL) are the main urinary ZEN biomarkers (Table 1). The proportion of α-ZEL in urine as a percentage of the sum of ZEN and α-ZEL level was 44.2% and 44.4% for groups D1 and D2, respectively. The fact that pigs mainly metabolize ZEN to α-ZEL is the main reason for ZEN sensitivity in pigs because the metabolite α-ZEL is more estrogenically active than ZEN [34]. Our findings are similar to the published literature to date (Supplementary Materials Table S1), although there are similarities in DON urinary concentrations—a high degree of variation within the group was observed in previous studies [17,21].

After feeding pigs more than 50 µg ZEN/kg feed, the metabolite beta-zearalenol (β-ZEL) was quantified as a ZEN biomarker in a few studies (Supplementary Materials Table S1). The metabolites zearalanone (ZAN), alpha- and beta-zearalanol (α-and β-ZAL), were only detected sporadically [17]. In our experiment, pigs were fed with a lower dose of ZEN, compared to the aforementioned studies, this explains why only ZEN and α-ZEL were quantified in pig urine.

The maximum level for ZEN in plasma was quantified 15–120 min after the administration of ZEN contaminated feed boluses [33,35]. That could explain why ZEN wasn’t found in the serum samples from experimental groups.

Neither ZEN nor its metabolites were found in the serum of the piglets and gilts (ingested up to 420 µg ZEN/kg feed) when the serum samples were analysed with the HPLC-FLD method. This was probably due to the relatively high limit of detection (LOD) for ZEN (1 ng/g for a sample weight of 5 g) [18,19]. Only traces of ZEN and α-ZEL were quantified in a dose-respond study—after exposure to feed contaminated with 50–420 µg ZEN/kg feed, and 170–420 µg ZEN/kg feed, respectively [17].

#### 2.2.3. Ochratoxin A Biomarkers

There were only a few experiments with pigs regarding OTA biomarkers. In a recent study with pigs OTA was quantified in urine samples at the following concentrations: 0.12 ± 0.05 ng/mL, 0.65 ± 0.22 ng/mL, 0.52 ± 0.15 ng/mL, and 0.36 ± 0.12 ng/mL after exposure to 1.43, 4.08, 4.96, and 11.72 µg/kg feed [21]. It is difficult to find a correlation between OTA intake and urinary OTA concentration from these data as a consequence—in order to compare those results with our data. Additionally, in our experiment, the pigs were exposed to higher doses of OTA (about two- and four-times the recommended EU level (Table 4)) from naturally contaminated feed—and in contrast to the aforementioned paper—feed boluses contaminated with mycotoxin extracts. In other studies, HPLC methods were applied for mycotoxin biomarker quantification [36].

The urine samples were analysed once for OTA at the end of the 5-month feeding period for OTA-contaminated diets, 1 week and 1 month after changing to the OTA-free diet. After feeding pigs with 130–790 µg/kg of OTA, mean urinary OTA levels of 52.37 ± 16.44 ng/mL were found [37]. The urine was only collected from four pigs from different OTA feeding groups. After one week on an OTA-free diet, the OTA was quantified in urine from 2 pigs—the mean concentration—15.08 ± 12.82 ng/mL. After one month on an OTA-free diet, the OTA couldn’t be quantified in the urine obtained from 2 pigs. Our results confirm that the OTA was quantified in urine till 2 weeks after changing the contaminated diet to the control one. Moreover, urine was collected from significantly more pigs (5 pigs for a group during 14 days).

At the time of the experiment, the metabolite ochratoxin alpha (OTα) still hadn’t been quantified in pig urine in contrast to human urine (which was frequently quantified), even in much higher concentrations (up to 20-times) than OTA [38,39]. Our results show, on average, about 2-times lower OTα levels than OTA in pig urine but indicate that OTα is an important OTA biomarker in pig urine (Table 1) and therefore it should be monitored together with OTA. Until recently, OTα was not included in monitoring pig urine biomarkers [16], leading to incomplete results.

OTA has frequently been quantified in human serum [40,41] in contrast to OTα—it was recently quantified in one study in 98% of blood samples [42].

Few animal studies (the last one was published in 2001) with pigs reported the serum levels of OTA after the oral exposure to the toxin. In a recent study, the pigs were exposed to 50 µg/kg feed (D1 group) and 500 µg/kg feed (D2 group) for 15 days. OTA was quantified in plasma samples at the following concentrations: 22.2 ± 2.6 ng/mL, 217.4 ± 25.1 ng/mL in the D1 and D2 groups. In this experiment, the basal diets were mixed with pure OTA standard solutions. That may explain why the OTA serum levels found in our experiment (naturally contaminated feed) were higher.

On one occasion, blood was collected from different slaughterhouses from different areas of Sweden. In the blood of pigs from 16.8% of herds higher OTA levels than 2 ng/mL were quantified [43]. In another study, the serum samples were analysed for OTA at the end of a 5-month feeding period of OTA-contaminated diets, 1 week and 1 month after changing to the OTA-free diet. Oral exposure to diets contaminated with 130–790 µg/kg of OTA resulted in mean serum OTA levels of 18.2 ± 4.76 ng/mL [37]. Serum samples were only collected from six pigs from different OTA feedings groups. After one week on an OTA-free diet, only traces of OTA were quantified in the serum from three pigs. After one month on an OTA-free diet, the OTA couldn’t be quantified in serum obtained from the three pigs. Our results confirm that OTA was quantified in serum until 2 weeks after changing the contaminated diet to the control one. Moreover, the serum was collected from significantly more pigs (four-times from five pigs from every group).

#### 2.2.4. Citrinine Biomarkers

Our results represent the first dose-response report concerning CIT urinary biomarkers in pigs. CIT and the metabolite dihydrocitrinone (DH-CIT) (quantified at similar levels as CIT) are suitable biomarkers of exposure in pigs.

CIT is often detected in pig feed and may impair animal health and performance [44,45]. According to recent data DH-CIT is much less toxic than CIT, suggesting that the production of this metabolite may be considered to be a detoxication reaction [46].

Recent studies have shown that CIT and its metabolite DH-CIT are often detected in human urine samples [47]. In the first human toxicokinetic study, the metabolite DH-CIT was found in the urine samples of volunteers at an approximately threefold higher levels than CIT. Therefore, the metabolite may be an important biomarker of mycotoxin exposure [48].

In the serum of the D2 group, lower concentrations of CIT were quantified (Table 2) as compared with urine and only traces of its metabolite DH-CIT. In the recent toxicokinetic analysis, CIT (10–100 ng/mL) as well as DH-CIT (0.3–0.7 ng/mL) were quantified in all plasma samples after the administration of boluses of 50 μg CIT/kg body weight [49], which is in accordance with the results of our study.

### 2.3. The Impact of the Duration of Exposure to Mycotoxins on the Biomarker Levels in Biological Matrices and the Kinetics of the Dissapearance of Mycotoxin Biomarkers

Our prolonged experiment (24-h urine samples were taken over the course of the 14 days of the experimental diets) which allowed us not only to select the most important mycotoxin biomarkers and determine their correlation with ingested feed but also to assess the impact of the experiment duration on biomarker levels (Table 3).

**Figure 1:**
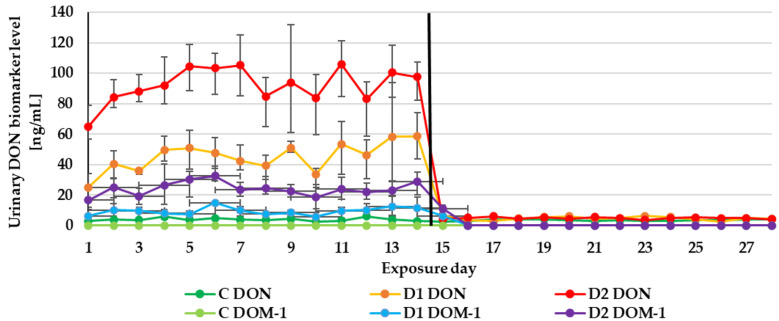
Mean urinary deoxynivalenol (DON) biomarker (DON and deepoxy-deoxynivalenol (DOM-1)) levels quantified during the experiment—14 days of experimental diets (C-control group, D1 and D2 group) and after substituting the contaminated diets for the control diet (day 15–28).

**Figure 2:**
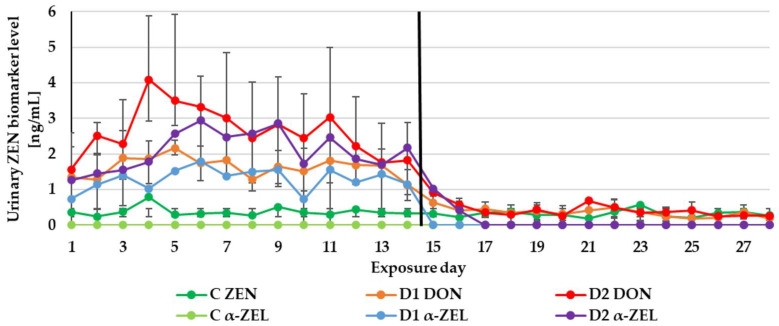
Mean urinary zearalenone (ZEN) biomarker (ZEN and alpha-zearalenol (α-ZEL)) levels quantified during the experiment—14 days of experimental diets (C-control group, D1 and D2 group) and after substituting the contaminated diets for the control diet (day 15–28).

**Figure 3:**
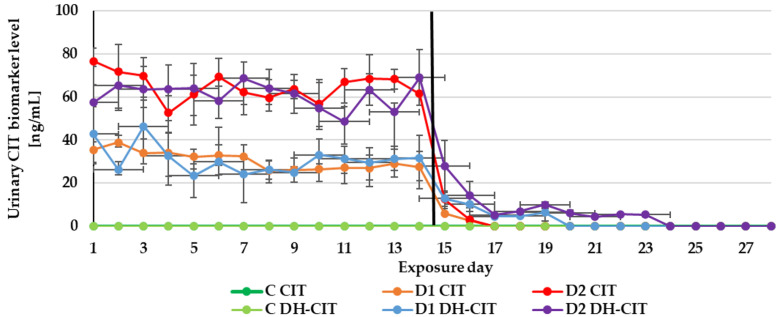
Mean urinary citrinine (CIT) biomarker (CIT and dihydrocitrinone (DH-CIT)) levels quantified during the experiment—14 days of experimental diets (C-control group, D1 and D2 group) and after substituting the contaminated diets for the control diet (day 15–28).

**Figure 4:**
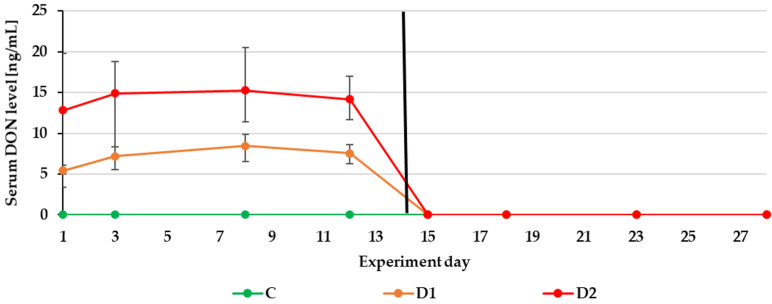
Mean serum DON levels quantified during the experiment—14 days of experimental diets (C-control group, D1 and D2 group) and after substituting the contaminated diets for the control diet (day 15–28).

**Figure 5:**
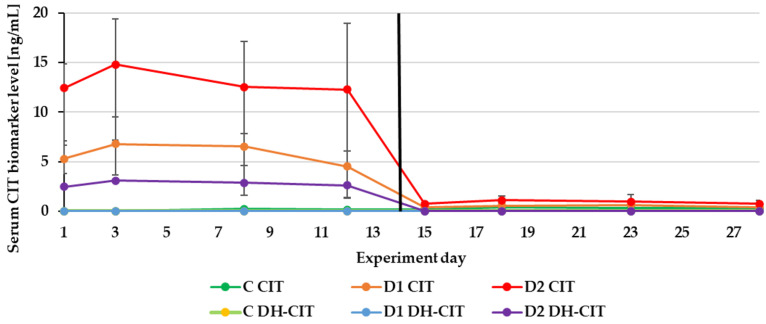
Mean urinary CIT biomarker (CIT and DH-CIT) levels quantified during the experiment—14 days of experimental diets (C-control group, D1 and D2 group) and after substituting the contaminated diets for the control diet (day 15–28).

The experiment duration didn’t influence the DON, ZEN and CIT biomarker levels in urine (Figure 1, Figure 2 and Figure 3) as well as the DON and CIT biomarker levels in serum (Figure 4 and Figure 5).

**Figure 6:**
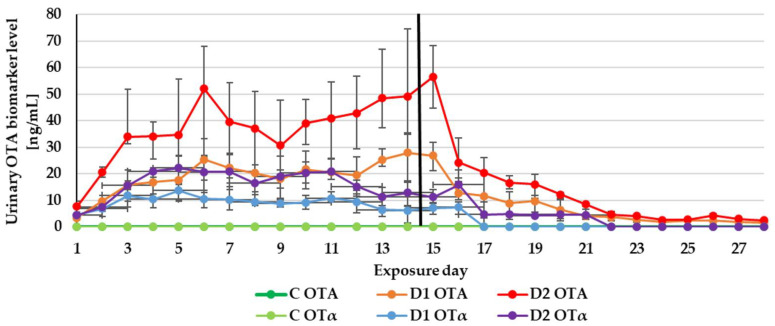
Mean urinary ochratoxin A (OTA) biomarker (OTA and ochratoxin alpha (OTα)) levels quantified during the experiment—14 days of experimental diets (C-control group, D1 and D2 group) and after substituting the contaminated diets for the control diet (day 15–28).

**Figure 7:**
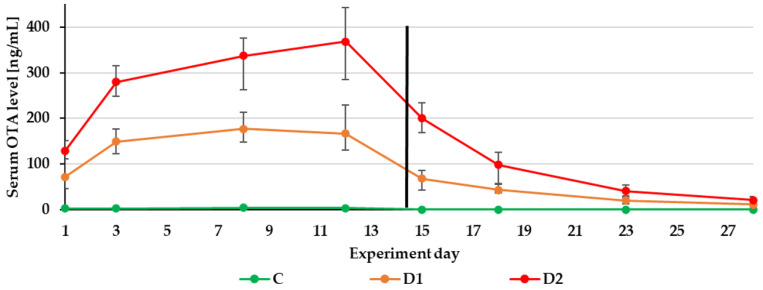
Mean serum OTA levels quantified during the experiment—14 days of experimental diets (C-control group, D1 and D2 group) and after substituting the contaminated diets for the control diet (day 15–28).

The impacts of the time of toxin exposure within the feeding groups were noticed for OTA in the urine and serum samples as well as OTα in the urine samples (Figure 6 and Figure 7). Therefore, in practice, the survey of OTA urinary biomarkers should last for a longer time—a minimum of three days—in order to observe the eventual changes in urinary OTA levels.

The design of our experiment (after two weeks the contaminated diets were substituted for the control) allowed for the assessment of the kinetic pathway of the disappearance of mycotoxin biomarkers from the biological matrices.

After substituting the contaminated feeds for the control (day 15), only traces of DON and ZEN could be quantified in the urine sample from the control or two experimental groups (D1, D2). Of the metabolites in the D2 group: DOM-1 could be quantified on day 15 and α-ZEL on day 15 and 16 (Figure 1 and Figure 2). This finding is in agreement with previous experimental results. The majority of ZEN and its metabolites (20 ± 11% of the administered dose) were excreted via urine production within the first 24 h after the application of contaminated feed boluses [15]. After the oral exposure to DON, about 85% of the dose was found in urine samples within 24 h [13]. As with urinary DON biomarkers, DON couldn’t be quantified in serum on day 15 (Figure 4).

In contrast to DON and ZEN, the OTA and CIT biomarkers were detected in urine and serum even after the completion of the experiment (Table 4). This finding complies with a previous pharmacokinetic study—the elimination half-lives of OTA and CIT in pigs were very long 72–120 h [50] and 17–26 h [49], respectively. This may be related to a higher degree of affinity towards porcine serum albumin.

## 3. Conclusions

Although to date, there have been few dose-response studies that feature DON and ZEN administration conducted with pigs, there were substantial differences in urinary biomarker concentration within and between the feeding groups in a majority of them. Biomarkers were rarely analysed in porcine serum. Additionally, there are significant differences in the results from experiments with pigs. This could be caused the use of contaminated feed or pure toxin. Moreover, in the majority of studies with pigs the urine samples were taken before slaughtering. This condition hindered the establishment of valuable relationships between the toxin levels in biological samples and feed. This is crucial because guidance values only exist for pig feed.

Our prolonged (14-day) experiment after the exposure of the pigs to naturally contaminated feed (what can better reflect real-life situations) could be very helpful in the establishment of guidance levels for mycotoxin biomarkers in biological matrices or to correlate it with existing feed guidance values. This is especially important for DON and ZEN, the concentrations of which, in contaminated feed were close to the EU guidance values and highly correlated with their urinary and serum biomarker levels. Additionally, the urinary levels of OTα, CIT and DH-CIT were reported for the first time herein. These findings could contribute to a better exposure assessment method for pigs with regard to OTA and CIT (important contaminants in pig feed).

Moreover, our study is the first report concerning the kinetic pathway of mycotoxin biomarkers in urine and serum samples for multiple mycotoxins. The prolonged presence and high concentrations of OTA biomarkers in the urine and serum samples demonstrate why the biomonitoring of mycotoxins in pigs is so important. Indeed, even if mycotoxins are not present in the feed samples, they can still be quantified in biological matrices. This finding could be very helpful in the assessment of the impact of a prolonged exposure to OTA on the health of pigs.

## 4. Materials and Methods

### 4.1. Chemicals and Reagents for the LC-MS/MS Analysis of Serum Biomarkers

Standard solutions of deoxynivalenol (DON), 3-acetyldeoxynivalenol (3-AcDON), 15-acetyldeoxynivalenol (15-AcDON), citrinin (CIT), diacetoxyscirpenol (DAS), zearalenone (ZEN), T-2 toxin (T-2), HT-2 toxin (HT-2), nivalenol (NIV), tentoxin (TEN), alternariol (AOH), alternariol monomethyl ether (AME) (100 μg/mL), deepoxy-deoxynivalenol (DOM-1), sterigmatocystin (STC), T-2 triol, ochratoxin A (OTA), ochratoxin alpha (OTα) (10 μg/mL) prepared in acetonitrile (ACN) and a standard solution of fumonisin B_1_ (FB_1_), fumonisin B_2_ (FB_2_) (50 μg/mL), hydrolysed fumonisin B_1_ (HFB_1_) (25 μg/mL) prepared in ACN:H_2_O (50:50, *v/v*) were purchased from Romer Labs Diagnostic (Tulln, Austria) and stored at 2–8 °C (except DOM-1—which was stored at ≤−15 °C). Aflatoxin B_1_ (AFB_1_), aflatoxin B_2_ (AFB_2_), aflatoxin G_1_ (AFG_1_), aflatoxin G_2_ (AFG_2_), aflatoxin M_1_ (AFM_1_), α-zearalenol (α-ZEL), β-zearalenol (β-ZEL), α-zearalanol (α-ZAL), β-zearalanol (β-ZAL), zearalanone (ZAN), enniatins (ENNs): enniatin A (ENA), enniatin B (ENB), enniatin A_1_ (ENA_1_) and enniatin B_1_ (ENB_1_) and beauvericin (BEA) were purchased in powder form (5 mg) from Sigma-Aldrich (Diegem, Belgium) and stored at ≤−15 °C. Dihydrocitrinone (DH-CIT) was purchased in powder form (1 mg) from AnalytiCon Discovery GmbH (Potsdam, Germany) and stored at ≤−16 °C.

Internal standards: U-[^13^C_17_]-AFLB_1_ (0.5 μg/mL), U-[^13^C_15_]-DON (25 μg/mL), U-[^13^C_20_]-OTA (10 μg/mL), U-[^13^C_24_]-T-2 (25 μg/mL), U-[^13^C_18_]-ZEN (25 μg/mL) were purchased from Romer Labs Diagnostic and stored at ≤−16 °C.

Beta-glucuronidases from *Escherichia coli* (*E.coli*, lyophilized powder, 1,000,000–5,000,000 units/g protein)) (Type IX-A) were obtained from Sigma–Aldrich (Darmstadt, Germany) and stored at 2–8 °C (with the exception of *E.coli*—stored at −20 °C).

Acetonitrile (ACN, analytical grade and LC-MS grade), methanol (MeOH, LC-MS grade), ethyl acetate (EtAc, HPLC grade), acetic acid were purchased from J.T. Baker (Avantor Performance Materials, Deventer, The Netherlands). Formic acid and ammonium acetate (LC-MS grade and analytical grade) and sodium chloride (NaCl, analytical grade) were from Sigma-Aldrich. Ultrapure water was produced using a Milli-Q system (Millipore, Bedford, MA, USA).

### 4.2. Preparation of Standard Mixtures for Serum Analysis

From the individual stock standard solutions, a standard mixture was prepared in acetonitrile and stored at −20 °C. A fresh standard mixture was prepared every month at the following concentrations: DON (200 ng/mL), 3-AcDON (40 ng/mL), 15-AcDON (80 ng/mL), DOM-1 (40 ng/mL), ZEN (2.5 ng/mL), α-ZEL (7.5 ng/mL), β-ZEL (10 ng/mL), α-ZAL (2.5 ng/mL), β-ZAL (300 ng/mL), ZAN (5 ng/mL), OTA (100 ng/mL), AFB_1_, AFB_2_, AFM_1_ (20 ng/mL), AFG_1_, AFG_2_ (50 ng/mL), T-2 (25 ng/mL), HT-2 (100 ng/mL), NIV (100 ng/mL), TEN (10 ng/mL), AOH (50 ng/mL), AME (5 ng/mL), CIT (5 ng/mL), DAS (20 ng/mL), STC (1 ng/mL), T-2 triol (25 ng/mL), OTα (25 ng/mL), HFB_1_ (100 ng/mL), DH-CIT (100 ng/mL), mix ENNs + BEA (10 ng/mL).

Additionally, an internal standard mixture was prepared from the individual stock internal standard solutions and stored at −20 °C. A fresh standard mixture was prepared every month at the following concentrations: U-[^13^C_17_]-AFB_1_ (0.01 μg/mL), U-[^13^C_15_]-DON (0.5 μg/mL), U-[^13^C_20_]-OTA (0.2 μg/mL), U-[^13^C_24_]-T-2 (0.5 μg/mL), U-[^13^C_18_]-ZEN (0.5 μg/mL).

### 4.3. Preparation of Experimental Diets

Feed was prepared from corn naturally contaminated with mycotoxins and rye—and artificially inoculated in the field experiment with DON-producing *Fusarium culmorum* strain (Table 5). Maize, rye and feed were analysed for the presence of mycotoxins with extract dilution following the LC-MS/MS method [51]. The control feed contained only traces of CIT, OTA. The concentration of mycotoxins in both experimental feeds along with the relationship with the European recommendations for pig feed [22] are shown in Table 5.

It is important to highlight that the RSD values of feed analysis were lower than 20% which shows the excellent homogeneity of the feed. The composition of feed, metabolizable energy and the analyses of selected ingredients of diets are shown in Supplementary Materials Table S4. The analytical composition of the feed is presented in Supplementary Materials Table S5.

### 4.4. Pig Trial

All of the experimental procedures involving animals were carried out in compliance with Polish legal regulations determining the terms and methods for performing experiments on animals (Opinion No. 42/2019 of the Local Ethics Committee for Animal Experimentation at the University of Warmia and Mazury in Olsztyn, Poland 28 May 2019).

The experiment was performed in the Department of Veterinary Prevention and Feed Hygiene at the Faculty of Veterinary Medicine of the University of Warmia and Mazury in Olsztyn (Poland) on 24 clinically healthy gilts purchased from a pig breeding farm in Jabłonowo (Pomorskie voivodeship, Poland). The animals were Polish Landrace × Polish Large White crossbreeds with an average initial body weight of 28.54 ± 1.98 kg (♂/♀, 12/12). The pigs were kept in individual metabolic cages with free access to water and were fed in the morning and evening under a restrictive feeding system. The lighting was natural through windows in the barn. The temperature was maintained between 22 and 26 °C. The animals were housed under these conditions for one week in order to acclimatize. During the experiment, body weight and feed intake were determined on a weekly basis. Feed intake during the experiment (per animal per day) was as follows: week 1–720 g, week 2–770 g, week 3–820 g, week 4–870 g.

The animals were randomly allocated to three groups of eight pigs. After the acclimatization period, over the next 14 days, every group received different feed—control feed (control group–C), and two experimental feeds: feed with a lower mycotoxin content (D1 group) and higher—about double the mycotoxin content in comparison to the D1 group (D2 group) (Table 5).

For the next 2 weeks (day 15–28), contaminated feed in groups D1 and D2 was substituted for the control in order to study the disappearance of mycotoxin biomarkers.

Serum samples were collected eight-times during the experiment in order to ensure the welfare of the pigs. A volume of 5 mL of blood was collected from cranial vena cava into sterile, 15 mL, polypropylene puncture blood collection tubes (Sarstedt, Nümbrecht, Germany) 3.5 h after feeding, on eight occasions during the experiment. The samples were located in a fridge, centrifuged after four hours (3.724× *g*, 10 min, 4 °C), and the serum was stored at ≤−15 °C.

Next, individual 24-h urine samples were also taken from each pig every morning from a bucket located under the metabolic cage. The samples were mixed thoroughly, and about 50 mL was collected in a sample tube. The samples were centrifuged (5000× *g*, 15 min, 4 °C) and stored at ≤−15 °C until analysis.

### 4.5. Mycotoxin Biomarkers LC-MS/MS Analysis

#### 4.5.1. Urine Samples Analysis

The urine samples were analysed in duplicate according to a recently developed and validated LC-MS/MS method [52]. Creatinine was analysed in every urine sample according to the developed HPLC method [31].

#### 4.5.2. Blood Sample Analysis—Method Development

The first step was to select a suitable matrix for analysis, the serum and plasma (*n* = 3) which were spiked at a medium QC level (Supplementary Materials Table S2) were analysed.

The extraction procedure was optimized using the serum samples (*n* = 3) spiked at a medium QC level (Supplementary Materials Table S2). Different extraction solvents (ACN, acidified ACN (0.1% HCOOH), MeOH, ACN: MeOH (1:1) and EtAc were tested.

The final optimized extraction procedure with acidified ACN (0.1% HCOOH) was used for method validation.

The optimum conditions obtained from the experimental design were applied: 250 µL of serum was transferred into an Eppendorf tube, followed by the addition of 750 µL ACN (0.1% HCOOH). An extraction was performed using a vortexer (2 × 30 s per sample), followed by centrifugation for 15 min at 14,000× *g*. Then, 800 µL of the supernatant was aspirated into a new tube, 10 µL of the internal standard mixture was added and evaporated to dryness under a gentle stream of nitrogen at 45 °C. Next, 100 µL of injection solvent, which contained 50% each eluent A and B was used to reconstitute the residue. After centrifugation for 10 min at 14,000× *g*, a 100 µL volume of this filtrate was placed into vials and used for LC-MS/MS analysis.

A matrix-matched calibration curve (QC samples, Supplementary Materials Table S2), together with a blank urine and a pure solvent control, were analysed for each batch of samples to ensure the reliability of the results. The analyte concentrations were determined through the use of isotopically labelled internal standards (IS).

The method was validated concerning the guidelines specified by the EMEA (2011) in terms of linearity, selectivity, sensitivity (LOD and LLOQ), accuracy, precision (intra- and inter-day variability), the matrix effect and carry-over. The method is described in detail for urine samples [52].

#### 4.5.3. LC-MS/MS Conditions

The analysis was carried out using an Agilent 1260 Infinity HPLC system (Agilent Technologies, Waldbronn, Germany) coupled to a QTRAP^®^6500 mass spectrometer (AB Sciex, Foster City, CA, USA). The detection of the analytes of interest was carried out using ESI ionization with Scheduled MRM (multiple reaction monitoring) detection mode parameters set to a window width of 60 s and a target scan time of 0.4 s in negative and positive ionization mode. Analyst^®^ software version 1.6.2. (AB Sciex) was used for data acquisition and processing. Additionally, MultiQuantTM 3.0.1 Software (AB Sciex) was used for data processing. LC-MS/MS conditions were developed recently and applied to urine analysis [52].

### 4.6. Statystical Analysis

The mean, standard deviation (SD) and relative standard deviation (RSD) of the results were calculated using Microsoft Excel 2020 software (Microsoft Corporation, Redmond, WA, USA). Data concerning mycotoxin intake and its respective levels in samples of urine and serum from the control and two experimental groups (D1 and D2) were subjected to a one-way ANOVA using the General Linear Model procedures of SAS^®^. The correlation between the mycotoxin intake and the concentrations of mycotoxin biomarker in the urine and serum samples was assessed using Pearson’s Correlation Coefficient (r). A Student *t*-test, with a normal approximation, was used to compare the correlation values. The statistical significance was accepted at *p* < 0.05.

## Figures and Tables

**Table 1:** Corellation coefficients (r) between mycotoxin intake (mg/kg feed) and the respective levels (mg/mL) in urine or plasma from pigs fed with mycotoxin-contaminated diets over the course of 14 days.

Mycotoxin Biomarkers	Urine		Serum	
	*r*	*p*	*r*	*p*
DON	0.999	<0.001	0.999	<0.001
DOM-1	0.978	<0.001	-	-
ZEN	0.987	<0.001	-	-
α-ZEL	0.966	<0.001	-	-
OTA	0.988	<0.001	0.999	<0.001
OTα	0.997	<0.001	-	-
CIT	0.998	<0.001	0.999	<0.001
DH-CIT	0.995	<0.001	-	-

DON—deoxynivalenol, DOM-1—deepoxy-deoxynivalenol, ZEN—zearalenone, α-ZEL—alpha-zearalenol, OTA—ochratoxin A, OTα—ochratoxin alpha, CIT—citrinine, DH-CIT—dihydrocitrinone.

**Table 2:** Urinary biomarkers quantified in control group (C) as well as the D1 and D2 group during the first 14 days of the experimental diets.

Urinary Biomarkers	LLOQ [ng/mL]	C Mean Level ± SD [ng/mL]	C Range [ng/mL]	D1 Mean Level ± SD [ng/mL]	D1 Range [ng/mL]	D2 Mean Level ± SD [ng/mL]	D2 Range [ng/mL]
DON	2.0	4.05 ± 0.83	2.11–7.27	45.3 ± 9.58	16.6–87.8	92.4 ± 11.6	51.5–127
DOM-1	6.0	<LLOQ	-	9.50 ± 2.51	2.06–20.1	24.2 ± 4.47	8.96–42.3
ZEN	0.1	0.38 ± 0.12	0.15–1.66	1.63 ± 0.29	0.49–2.96	2.63 ± 0.71	0.55–6.51
α-ZEL	0.4	<LLOQ	-	1.29 ± 0.31	0.43–2.46	2.10 ± 0.55	0.68–4.42
OTA	1.5	<LLOQ	-	18.8 ± 6.40	2.38–39.1	36.5 ± 11.6	5.76–77.2
OTα	4.0	<LLOQ	-	9.48 ± 2.62	3.87–17.8	16.2 ± 5.49	4.04–28.5
CIT	1.5	<LLOQ	-	30.6 ± 4.17	20.1–44.9	64.9 ± 6.45	44.7–89.5
DH-CIT	4	<LLOQ	-	31 ± 6.63	13.9–59.9	61.1 ± 5.94	40.5–81.3

**Table 3:** Serum biomarkers quantified in the control group (C) as well as the D1 and D2 group during the first 14 days of the experimental diets.

Serum Biomarkers	LLOQ [ng/mL]	C Mean Level ± SD [ng/mL]	C Range [ng/mL]	D1 Mean Level ± SD [ng/mL]	D1 Range [ng/mL]	D2 Mean Level ± SD [ng/mL]	D2 Range [ng/mL]
DON	2	<LLOQ	-	7.13 ± 1.26	5.42–8.42	14.3 ± 1.06	12.8–15.3
OTA	2	<LLOQ	-	141 ± 47.9	71.5–177	278 ± 106	129–368
OTα	0.5	<LLOQ	-	<LLOQ	-	0.69 ± 0.10	0.51–1.10
CIT	0.5	0.19 ± 0.04	<LLOQ-0.22	5.77 ± 1.07	4.50–6.78	13.03 ± 1.20	12.3–14.8
DH-CIT	0.1	<LLOQ	-	<LLOQ	-	2.76 ± 0.55	2.02–3.83

**Table 4:** The most important urinary and serum biomarkers, the kinetic pathway of their disappearance and the impact of the exposure duration on biomarker levels in urine and serum samples (the biomarkers found at the highest concentration are highlighted).

Mycotoxin Ingested	Urinary Biomarkers	Metabolite/ Mycotoxin +Metabolite [%]	Kinetics of the Disappearance of Urinary Biomarkers	Serum Biomarkers	Kinetic of the Disappearance of Serum Biomarkers	Impact of Experiment Duration on Biomarker Levels
DON	**DON** DOM-1	18	<48 h	DON	<48 h	-
ZEN	**ZEN** α-ZEL	44	<72 h	n.d.	-	-
OTA	OTA OTα	31	>14 days >6 days	**OTA**	>14 days	+
CIT	**CIT** DH-CIT	50	>2 days >9 days	CIT	>14 days	-

**Table 5:** Mycotoxin levels in corn, rye which were the ingredients of the pig control feed and two experimental feeds (for D1 and D2 group) with the European limits.

Mycotoxin Concentration [µg/kg]	Corn	Rye	EU Guidance Value	Control Group	RSD [%]	D1 Group	RSD [%]	D2 Group	RSD [%]
**DON**	1200 ± 103	4200 ± 234	900	<LLOQ	-	559 ± 23.0	4.11	1126 ± 91.4	8.12
**ZEN**	76.4 ± 11.2	6.34 ± 3.24	100 *	<LLOQ	-	15.8 ± 2.11	13.3	34.7 ± 4.34	12.5
			250 **						
**OTA**	535 ± 32.4	n.d.	50	4.46 ± 0.48	10.8	114 ± 8.38	7.33	226 ± 21.8	9.68
**CIT**	207 ± 24.8	n.d.	-	7.50 ± 1.05	14.1	35.7 ± 5.02	14.1	71.3 ± 8.28	11.6

* Complementary and complete feedstuffs for piglets and gilts (young sows); ** Complementary and complete feedstuffs for sows and fattening pigs.

## Data Availability

Not applicable.

## Supplementary Materials

The following are available online at https://www.mdpi.com/article/10.3390/toxins13110750/s1, Table S1. Recent (after the year 2000) dose-respond study with DON and ZEN administration in pigs; Table S2. The concentration of analytes in QC samples (spiked serum) and performance characteristics of the developed method (extraction recovery (R_E_), apparent recovery (R_A_), matrix effect (SSE) for six different serum samples and the CV of the IS-normalized SSE (CV(SSE)); Table S3. Values of accuracy and precision: (a) Within-Day accuracy and precision (*n* = 6), (b) Between-Day accuracy and precision (*n* = 6), (c) Two different batch of samples—accuracy and precision (*n* = 6); Table S4. Composition of feed, metabolizable energy and analyses of selected ingredients of diets, Mix parameters in 1 kg; Table S5. Analytical composition of the feed, Mix parameters in 1 kg.

## References

[1] European Parliament and the Council of the, EU (2002). Directive of The European Parliament and of the Council of 7 May 2002 on undesirable substances in animal feed 2002/32. Off. J. Eur. Communities.

[2] Pierron A., Alassane-Kpembi I., Oswald I. (2016). Impact of mycotoxin on immune response and consequences for pig health. Anim. Nutr..

[3] Bryden W.L. (2012). Mycotoxin contamination of the feed supply chain: Implications for animal productivity and feed security. Anim. Feed. Sci. Technol..

[4] Swindle M.M., Makin A., Herron A.J., Clubb F.J., Frazier K.S. (2011). Swine as Models in Biomedical Research and Toxicology Testing. Vet. Pathol..

[5] Gasthuys E., Vandecasteele T., De Bruyne P., Walle J.V., De Backer P., Cornillie P., Devreese M., Croubels S. (2016). The Potential Use of Piglets as Human Pediatric Surrogate for Preclinical Pharmacokinetic and Pharmacodynamic Drug Testing. Curr. Pharm. Des..

[6] Pinotti L., Ottoboni M., Giromini C., Dell’Orto V., Cheli F. (2016). Mycotoxin Contamination in the EU Feed Supply Chain: A Focus on Cereal Byproducts. Toxins.

[7] European Commission (EC) (2006). Commission Recommendation of 17 August 2006 on the presence of deoxynivalenol, zearalenone, ochratoxin A, T-2 and HT-2 and fumonisins inproducts intended for animal feeding. Off. J. Eur. Union.

[8] Zain M.E. (2011). Impact of mycotoxins on humans and animals. J. Saudi Chem. Soc..

[9] Guo W.R., Ou S.X., Long W.P., Wei Z., Yan X., Yu L. (2015). Simultaneous Detection Method for Mycotoxins and their Metabolites in Animal Urine by Using Impurity Adsorption Purification followed by Liquid Chromatography-Tandem Mass Detection. J. Chromatogr. Sep. Tech..

[10] Song S., Ediage E.N., Wu A., De Saeger S. (2013). Development and application of salting-out assisted liquid/liquid extraction for multi-mycotoxin biomarkers analysis in pig urine with high performance liquid chromatography/tandem mass spectrometry. J. Chromatogr. A.

[11] Thieu N.Q., Pettersson H. (2009). Zearalenone, deoxynivalenol and aflatoxin B1 and their metabolites in pig urine as biomarkers for mycotoxin exposure. Mycotoxin Res..

[12] Lauwers M., Croubels S., Letor B., Gougoulias C., Devreese M. (2019). Biomarkers for Exposure as A Tool for Efficacy Testing of A Mycotoxin Detoxifier in Broiler Chickens and Pigs. Toxins.

[13] Nagl V., Woechtl B., Schwartz-Zimmermann H., Hennig-Pauka I., Moll W.-D., Adam G., Berthiller F. (2014). Metabolism of the masked mycotoxin deoxynivalenol-3-glucoside in pigs. Toxicol. Lett..

[14] Eriksen G.S., Pettersson H., Lindberg J.E. (2003). Absorption, metabolism and excretion of 3-acetyl DON in pigs. Arch. Anim. Nutr..

[15] Binder S.B., Schwartz-Zimmermann H.E., Varga E., Bichl G., Michlmayr H., Adam G., Berthiller F. (2017). Metabolism of Zearalenone and Its Major Modified Forms in Pigs. Toxins.

[16] Gambacorta L., Olsen M., Solfrizzo M. (2019). Pig Urinary Concentration of Mycotoxins and Metabolites Reflects Regional Differences, Mycotoxin Intake and Feed Contaminations. Toxins.

[17] Brezina U., Rempe I., Kersten S., Valenta H., Humpf H.-U., Dänicke S. (2014). Diagnosis of intoxications of piglets fed with Fusarium toxin-contaminated maize by the analysis of mycotoxin residues in serum, liquor and urine with LC-MS/MS. Arch. Anim. Nutr..

[18] Döll S., Dänicke S., Ueberschär K.-H., Valenta H., Schnurrbusch U., Ganter M., Klobasa F., Flachowsky G. (2003). Effects of graded levels of Fusarium toxin contaminated maize in diets for female weaned piglets. Arch. Anim. Nutr..

[19] Dänicke S., Brüssow K.-P., Valenta H., Ueberschär K.-H., Tiemann U., Schollenberger M. (2005). On the effects of graded levels of Fusarium toxin contaminated wheat in diets for gilts on feed intake, growth performance and metabolism of deoxynivalenol and zearalenone. Mol. Nutr. Food Res..

[20] Thanner S., Czeglédi L., Schwartz-Zimmermann H.E., Berthiller F., Gutzwiller A. (2016). Urinary deoxynivalenol (DON) and zearalenone (ZEA) as biomarkers of DON and ZEA exposure of pigs. Mycotoxin Res..

[21] Gambacorta S., Solfrizzo H., Visconti A., Powers S., Cossalter A., Pinton P., Oswald I. (2013). Validation study on urinary biomarkers of exposure for aflatoxin B1, ochratoxin A, fumonisin B1, deoxynivalenol and zearalenone in piglets. World Mycotoxin J..

[22] EFSA Panel on Contaminants in the Food Chain (CONTAM) (2012). Scientific Opinion on the risks for public and animal health related to the presence of citrinin in food and feed. EFSA J..

[23] Schrenk D., Bodin L., Chipman J.K., Del Mazo J., Grasl-Kraupp B., Hogstrand C., Hoogenboom L.R., Leblanc J., Nebbia C.S., EFSA Panel on Contaminants in the Food Chain (CONTAM) (2020). Risk assessment of ochratoxin A in food. EFSA J..

[24] Sun Y., Zhang G., Zhao H., Zheng J., Hu F., Fang B. (2014). Liquid chromatography–tandem mass spectrometry method for toxicokinetics, tissue distribution, and excretion studies of T-2 toxin and its major metabolites in pigs. J. Chromatogr. B.

[25] Devreese M., De Baere S., De Backer P., Croubels S. (2012). Quantitative determination of several toxicological important mycotoxins in pig plasma using multi-mycotoxin and analyte-specific high performance liquid chromatography–tandem mass spectrometric methods. J. Chromatogr. A.

[26] De Baere S., Osselaere A., Devreese M., Vanhaecke L., De Backer P., Croubels S. (2012). Development of a liquid–chromatography tandem mass spectrometry and ultra-high-performance liquid chromatography high-resolution mass spectrometry method for the quantitative determination of zearalenone and its major metabolites in chicken and pig plasma. Anal. Chim. Acta.

[27] Devreese M., De Baere S., De Backer P., Croubels S. (2013). Quantitative determination of the *Fusarium* mycotoxins beauvericin, enniatin A, A1, B and B1 in pig plasma using high performance liquid chromatography–tandem mass spectrometry. Talanta.

[28] Broekaert N., Devreese M., De Mil T., Fraeyman S., De Baere S., De Saeger S., De Backer P., Croubels S. (2014). Development and validation of an LC–MS/MS method for the toxicokinetic study of deoxynivalenol and its acetylated derivatives in chicken and pig plasma. J. Chromatogr. B.

[29] Lauwers M., De Baere S., Letor B., Rychlik M., Croubels S., Devreese M. (2019). Multi LC-MS/MS and LC-HRMS Methods for Determination of 24 Mycotoxins including Major Phase I and II Biomarker Metabolites in Biological Matrices from Pigs and Broiler Chickens. Toxins.

[30] Brezina U., Valenta H., Rempe I., Kersten S., Humpf H.-U., Dänicke S. (2014). Development of a liquid chromatography tandem mass spectrometry method for the simultaneous determination of zearalenone, deoxynivalenol and their metabolites in pig serum. Mycotoxin Res..

[31] Tkaczyk A., Jedziniak P. (2020). Dilute-and-Shoot HPLC-UV Method for Determination of Urinary Creatinine as a Normalization Tool in Mycotoxin Biomonitoring in Pigs. Molecules.

[32] Pestka J.J. (2007). Deoxynivalenol: Toxicity, mechanisms and animal health risks. Anim. Feed. Sci. Technol..

[33] Fleck S.C., Churchwell M.I., Doerge D.R. (2017). Metabolism and pharmacokinetics of zearalenone following oral and intravenous administration in juvenile female pigs. Food Chem. Toxicol..

[34] Malekinejad H., Maas-Bakker R., Fink-Gremmels J. (2006). Species differences in the hepatic biotransformation of zearalenone. Vet. J..

[35] Catteuw A., Broekaert N., De Baere S., Lauwers M., Gasthuys E., Huybrechts B., Callebaut A., Ivanova L., Uhlig S., De Boevre M. (2019). Insights into In Vivo Absolute Oral Bioavailability, Biotransformation, and Toxicokinetics of Zearalenone, α-Zearalenol, β-Zearalenol, Zearalenone-14-glucoside, and Zearalenone-14-sulfate in Pigs. J. Agric. Food Chem..

[36] Blank R., Wolffram S. (2004). Alkalinization of Urinary pH Accelerates Renal Excretion of Ochratoxin A in Pigs. J. Nutr..

[37] Stoev S., Vitanov S., Anguelov G., Petkova-Bocharova T., Creppy E. (2001). Experimental Mycotoxic Nephropathy in Pigs Provoked by a Diet Containing Ochratoxin A and Penicillic Acid. Vet. Res. Commun..

[38] Ali N., Muñoz K., Degen G.H. (2017). Ochratoxin A and its metabolites in urines of German adults—An assessment of variables in biomarker analysis. Toxicol. Lett..

[39] Duarte S.C., Pena A., Lino C. (2011). Human ochratoxin A biomarkers—From exposure to effect. Crit. Rev. Toxicol..

[40] Märtlbauer E., Usleber E., Dietrich R., Schneider E. (2009). Ochratoxin A in human blood serum—Retrospective long-term data. Mycotoxin Res..

[41] Coronel M.B., Sanchis V., Ramos A.J., Marin S. (2010). Review. Ochratoxin A: Presence in Human Plasma and Intake Estimation. Food Sci. Technol. Int..

[42] Ali N., Hossain K., Degen G.H. (2017). Blood plasma biomarkers of citrinin and ochratoxin A exposure in young adults in Bangladesh. Mycotoxin Res..

[43] Hult K., Hökby E., Gatenbeck S., Rutqvist L. (1980). Ochratoxin A in blood from slaughter pigs in Sweden: Use in evaluation of toxin content of consumed feed. Appl. Environ. Microbiol..

[44] Arroyo-Manzanares N., Rodríguez-Estévez V., Arenas-Fernández P., García-Campaña A.M., Gámiz-Gracia L. (2019). Occurrence of Mycotoxins in Swine Feeding from Spain. Toxins.

[45] Aland A., Madec F. (2009). Sustainable Animal Production.

[46] Föllmann W., Behm C., Degen G.H. (2014). Toxicity of the mycotoxin citrinin and its metabolite dihydrocitrinone and of mixtures of citrinin and ochratoxin A in vitro. Arch. Toxicol..

[47] Huybrechts B., Martins J.C., Debongnie P., Uhlig S., Callebaut A. (2014). Fast and sensitive LC–MS/MS method measuring human mycotoxin exposure using biomarkers in urine. Arch. Toxicol..

[48] Ali N., Blaszkewicz M., Degen G.H. (2014). Occurrence of the mycotoxin citrinin and its metabolite dihydrocitrinone in urines of German adults. Arch. Toxicol..

[49] Meerpoel C., Vidal A., Huybrechts B., Tangni E.K., De Saeger S., Croubels S., Devreese M. (2020). Comprehensive toxicokinetic analysis reveals major interspecies differences in absorption, distribution and elimination of citrinin in pigs and broiler chickens. Food Chem. Toxicol..

[50] Dietrich D., Heussner A.H., O’Brien E. (2005). Ochratoxin A: Comparative pharmacokinetics and toxicological implications (experimental and domestic animals and humans). Food Addit. Contam..

[51] Jedziniak P., Panasiuk Ł., Pietruszka K., Posyniak A. (2019). Multiple mycotoxins analysis in animal feed with LC-MS/MS: Comparison of extract dilution and immunoaffinity clean-up. J. Sep. Sci..

[52] Tkaczyk A., Jedziniak P. (2021). Development of a multi-mycotoxin LC-MS/MS method for the determination of biomarkers in pig urine. Mycotoxin Res..

