# Supplementary material for: Biomarkers of Deoxynivalenol, Citrinin, Ochratoxin A and Zearalenone in Pigs after Exposure to Naturally Contaminated Feed Close to Guidance Values
Source: Toxins (Basel). 2021 Oct 22;13(11):750. doi: 10.3390/toxins13110750 (PMC8625168; doi:10.3390/toxins13110750)
Supplement: Supplementary file 1 [file toxins-13-00750-s001.zip › toxins-1342382-supplementary.pdf]

toxins

MDPI

Supplementary Materials: Biomarkers of Deoxynivalenol, Citrinin, Ochratoxin A and Zearalenone in Pigs after Exposure to Naturally Contaminated Feed Close to Guidance Values

Agnieszka Tkaczyk, Piotr Jedziniak, Łukasz Zielonka, Michał Dąbrowski, Piotr Ochodzki and Adrianna Rudawska

Table S1. Recent (after the year 2000) dose-response study with DON and ZEN administration in pigs.

Dose DON [μg/kg]	DON urinary biomarkers	Biomarkers Level [ng/mL]	DON serum biomarkers	Biomarkers Level [ng/mL]	Dose ZEN [μg/kg]	ZEN urinary biomarkers	Biomarker Level [ng/mL]	ZEN serum biomarkers	Biomarkers Level [ng/mL]	Experiment time	Feed type	Number and type of Pig	Urine/ serum analysis	Ref.
30	DON<br>DOM-1	68.3* (18.6 – 231)<br>20.4 (5.3-171)	DON<br>DOM-1	0.5 (0-1.4)<br>0	10	ZEN<br>α-ZEL<br>β-ZEL<br>ZAN<br>α-ZAL	3.4* (0.5-11.4)<br>0.6 (<LOQ-35.2)<br><LOQ<br><LOQ<br><LOQ	ZEN<br>α-ZEL	0<br>0	29 days	contaminated maize	125 female weaned piglets	LC-MS/MS	[17]
590		524 (50.8-1070)<br>43.4 (1.8-140)		4.9 (2.7-7.9)<br>0	50		25.9 (1.0-82.0)<br>9.7 (0-22.7)<br><LOQ<br><LOQ<br><LOQ		0-0.1<br>0					
1270		1065 (96.2-2120)<br>70.3 (1.6-336)		8.6 (4.6-15.9)<br>0	80		51.7 (1.1.-122)<br>18.6 (0-63.2)<br>1.4 (<LOQ-18.2)<br>0.4 (<LOQ-1.4)<br><LOQ		0-0.2<br>0					
2010		1850 (288-4050)<br>118 (1.2-513)		15.2 (8.1-24.9)<br>2.9 (0-4.0)	170		79 (9.5-237)<br>35.1 (2.3-89.2)<br>10.2 (<LOQ-42.1)<br>0.8 (<LOQ-2.3)<br><LOQ		0-0.3<br>0-0.8					
4520		2680 (244-4990)<br>262 (0.7-979)		24.4 (9.9-42.8)<br>4.5 (0-7.4)	290		136 (7.7-327)<br>57.2 (2.4-122)<br>15.1 (0-42.9)<br>1.7 (0-3.4)<br>0.5 (0-0.7)		0-0.5<br>0-1.6					
200	DON<br>DOM-1	56**<br><LOD	DON	<LOQ	10	ZEN<br>α-ZEL	2.7**<br>1.5	-	-	37 days	contaminated maize	100 female piglets	HPLC-DAD	[18]
800		162<br><LOD		1 (<LOQ-4)	60		34.8<br>10.2							
1000		360<br>14		5 (<LOQ-12)	150		31.5<br>9.9							
1900		246<br>14		6 (<LOQ-13)	220		64.1<br>20.3							
3900		380<br>19		11 (6-19)	420		74.7<br>27.7							

Table S1. *Cont.*

Dose DON [μg/kg]	DON urinary biomarkers	Biomarker Level [ng/mL]	DON serum biomarkers	Biomarkers Level [ng/mL]	Dose ZEN [μg/kg]	ZEN urinary biomarkers	Biomarker Level [ng/mL]	ZEN serum biomarkers	Biomarkers Level [ng/mL]		Experiment time	Feed type	Number and type of Pig	Urine/ serum analysis
210	DON<br>DOM-1	299**<br>29	DON<br>DOM-1	1**<br>0.1	4	ZEN<br>α-ZEL	10.4<br>16.4	-	-	35 days	contaminated wheat	12 – 180 days old gilts	HPLC-DAD	[19]
3070		690<br>150		4.1<br>1.3	88		16.7<br>18.3							
6100		804<br>177		14.3<br>2.8	235		15.4<br>22.1							
9570		1572<br>289		21.6<br>4.1	358		43.1<br>57.9							
1110	-	-	-	-	350	-	-	-	-	28 days	contaminated maize	24 castrated male pigs	LC-MS/MS	[20]
2320					720									
3700					1680									
5000					2360									
63.58	DON<br>DOM-1	80.55** ± 28.96 ***<br>1.19 ± 1.38	-	-	6.04	ZEN<br>α-ZEL<br>β-ZEL	11.63 ± 7.52<br>3.60 ± 3.70<br>nd	-	-	3 days	feed boluses fortified with pure cultures	16 weaned piglets	LC-MS/MS	[21]
181.51		125.06 ± 41.64<br>3.36 ±1.35			17.23		23.06 ± 11.42<br>6.76 ± 4.43<br>nd							
214.36		305.94 ± 143.17<br>3.44 ± 4.14			21.13		15.76 ± 9.62<br>5.24 ± 3.29<br>nd							

Table S2. The concentration of analytes in QC samples (spiked serum) and the performance characteristics of the developed method (extraction recovery (R_E), apparent recovery (R_A), matrix effect (SSE) for six different serum samples and the CV of the IS-normalized SSE (CV(SSE)).

Analyte	LLOQ [ng/mL]	low QC [ng/mL]	medium QC [ng/mL]	high QC [ng/mL]	R_E [%]	R_A [%]	SSE [%]	CV (SSE) [%]
**CIT**	0.1	0.2	0.8	2	78.0	128	131	9.70
**α-ZEL**	0.15	0.3	1.2	3	73.6	61.2	84.6	4.76
**β-ZEL**	0.2	0.4	1.6	4	70.5	95.5	90.6	8.94
**ZEN**	0.05	0.1	0.4	1	85.6	69.5	94.2	7.96
**β-ZAL**	0.06	0.12	0.48	1.2	77.0	64.5	94.5	10.9
**α-ZAL**	0.05	0.1	0.4	1	72.9	61.4	88.1	8.02
**ZAN**	0.1	0.2	0.8	2	76.1	71.5	92.3	8.36
**NIV**	2	4	16	40	62.2	70.6	93.0	3.40
**DOM-1**	0.8	1.6	6.4	16	57.3	79.0	128	5.09
**DON**	2	4	16	40	76.4	104	151	5.76
**AFB_1**	0.4	0.8	3.2	8	76.0	103	150	11.1
**AFB_2**	0.4	0.8	3.2	8	69.2	56.7	76.8	6.18
**STC**	0.02	0.04	0.16	0.4	70.2	71.4	101	7.64
**$AFLM_1$**	0.4	0.8	3.2	8	71.9	72.1	106	6.41
**AFG_1**	2	4	16	40	68.2	56.5	77.1	6.20
**AFG_2**	2	4	16	40	50.7	39.2	70.6	12.1
**15-AcDON**	1.6	3.2	12.8	32	52.8	43.9	73.5	9.49
**3-AcDON**	0.8	1.6	6.4	16	60.0	75.4	171	9.63
**DAS**	0.4	0.8	3.2	8	63.9	86.0	177	7.43
**OTA**	2	4	16	40	73.9	64.5	98.4	11.6
**HT-2**	2	4	16	40	79.3	90.9	140	9.40
**T-2**	0.5	1	4	10	70.0	71.4	103	9.03
**ENB**	0.2	0.4	1.6	4	71.4	66.1	89.5	8.17
**ENB_1**	0.2	0.4	1.6	4	71.0	85.2	139	7.64
**ENA_1**	0.2	0.4	1.6	4	65.8	79.3	142	13.0
**ENA**	0.2	0.4	1.6	4	67.72	99.6	152	15.3
**BEA**	0.2	0.4	1.6	4	72.12	93.9	227	14.5
**AOH**	0.5	1	4	10	72.55	84.1	157	17.0
**AME**	0.05	0.1	0.4	1	67.58	67.6	99.2	5.19
**TEN**	0.1	0.2	0.8	2	65.29	59.0	90.5	12.3
**OTα**	0.5	1	4	10	79.68	75.3	85.0	8.91
**HFB_1**	2	4	16	40	71.50	57.9	98.0	13.7
**DH-CIT**	2	4	16	40	76.48	38.9	56.3	16.2
**T-2 triol**	0.5	1	4	10	70.14	49.2	71.3	16.3
**FB_2**	4	8	32	80	68.87	51.4	92.3	13.2
**FB_1**	4	8	32	80	56.93	61.3	74.1	6.04

Table S3. Validation results: values of accuracy and precision.

a) Within-Day accuracy and precision (n=6)

Analyte	Theoretical Concentration LLOQ		Theoretical Concentration low QC		Theoretical Concentration medium QC		Theoretical Concentration high QC	
	Accuracy [%]	Precision (RSD [%])	Accuracy [%]	Precision (RSD [%])	Accuracy [%]	Precision (RSD [%])	Accuracy [%]	Precision (RSD [%])
**CIT**	101	9.51	66.0	10.24	86.3	13.7	93.5	12.3
**α-ZEL**	90.0	14.4	112	8.43	102	12.5	94.5	12.2
**β-ZEL**	71.9	8.35	101	14.6	103	12.1	103	10.3
**ZEN**	91.8	9.13	91.2	6.09	106	6.34	91.4	10.5
**β-ZAL**	89.3	11.2	96.7	9.07	95.2	5.67	85.3	11.9
**α-ZAL**	87.8	9.87	100	8.49	104	8.07	98.8	9.28
**ZAN**	83.0	8.59	102	12.5	100	16.2	96.3	8.34
**NIV**	90.7	15.3	89.7	12.0	102	10.2	87.5	6.94
**DOM-1**	90.3	7.11	91.1	5.52	79.3	4.93	72.2	8.91
**DON**	91.8	4.19	97.1	6.10	88.8	2.44	88.5	6.66
**AFB_1**	108	15.0	96.8	12.8	103	7.01	97.1	11.5
**AFB_2**	103	7.70	103	3.91	105	11.2	102	11.5
**STC**	91.5	4.56	98.1	4.51	93.1	9.59	84.0	7.32
**$AFLM_1$**	113	11.3	105	10.8	108	10.2	115	8.53
**AFG_1**	106	5.63	101	6.95	110	14.8	112	4.25
**AFG_2**	113	9.74	100	14.1	103	15.1	106	8.95
**15-AcDON**	84.9	9.70	87.4	8.14	72.7	10.9	67.5	8.25
**3-AcDON**	87.8	10.5	100	7.66	83.1	5.51	78.2	11.3
**DAS**	95.5	15.1	92.8	9.55	97.2	7.59	85.4	6.22
**OTA**	102	7.73	102	5.41	105	8.23	86.2	5.49
**HT-2**	101	19.1	99.9	9.42	104	11.4	99.5	5.59
**T-2**	96.0	14.1	93.8	12.5	96.7	11.9	88.8	8.71
**ENB**	82.2	11.8	86.1	7.69	94.4	4.24	88.3	7.53
**ENB_1**	106	16.4	93.3	9.58	102	6.86	93.5	13.4
**ENA_1**	104	8.03	98.8	11.4	97.4	8.25	83.7	5.90
**ENA**	85.0	15.9	94.4	8.80	97.5	6.16	87.3	6.42
**BEA**	106	12.1	83.4	15.3	89.2	10.1	79.6	11.1
**AOH**	87.2	5.95	88.5	4.02	94.9	7.41	81.0	6.29
**AME**	86.1	10.8	99.3	5.80	89.6	11.3	82.1	10.1
**TEN**	80.2	11.4	89.3	5.39	111	11.0	105	12.7
**OTα**	94.1	4.18	95.0	3.71	89.2	10.5	79.4	6.30
**HFB_1**	91.3	8.42	102	5.12	108	6.16	92.3	6.62
**DH-CIT**	95.6	10.8	90.3	10.7	85.6	13.6	91.5	12.6
**T-2 triol**	91.5	15.6	77.8	10.7	103	11.3	105	7.53
**FB_2**	108	8.75	113	8.73	112	9.04	93.3	7.02
**FB_1**	89.9	15.3	107	14.6	111	12.8	93.6	11.0

b) Between-Day accuracy and precision (n=6)

Analyte	Theoretical Concentration LLOQ		Theoretical Concentration low QC		Theoretical Concentration medium QC		Theoretical Concentration high QC	
	Accuracy [%]	Precision (RSD [%])	Accuracy [%]	Precision (RSD [%])	Accuracy [%]	Precision (RSD [%])	Accuracy [%]	Precision (RSD [%])
**CIT**	97.1	15.9	93.8	13.4	104	13.11	101.06	15.0
**α-ZEL**	82.7	19.4	113	11.7	108	13.48	96.37	9.73
**β-ZEL**	90.3	18.8	98.5	12.4	102	12.64	99.39	13.3
**ZEN**	94.8	7.33	96.5	10.3	107	9.79	97.69	10.5
**β-ZAL**	93.1	12.0	105	10.7	104	9.56	98.43	13.3
**α-ZAL**	89.1	11.0	104	10.4	108	7.63	96.95	10.4
**ZAN**	87.1	11.1	103	10.7	103	12.55	98.85	8.39
**NIV**	96.5	10.8	96.9	10.5	107	10.19	97.72	9.55
**DOM-1**	93.5	8.66	99.2	8.82	105	15.24	96.23	14.8
**DON**	97.7	4.78	100	6.70	103	9.85	98.88	12.8
**AFB_1**	96.5	13.9	96.7	9.35	108	8.24	97.36	10.2
**AFB_2**	93.8	7.74	98.9	8.91	107	9.37	97.50	11.0
**STC**	93.4	5.14	104	7.31	104	11.51	98.18	13.0
**$AFLM_1$**	95.5	18.0	102	11.4	104	12.58	98.39	13.1
**AFG_1**	103	13.5	93.9	15.6	105	11.66	98.51	11.7
**AFG_2**	103	13.8	95.0	13.4	103	11.66	99.36	11.1
**15-AcDON**	97.7	14.7	103	13.1	98.8	13.66	100.26	12.4
**3-AcDON**	92.1	12.7	105	9.83	103	15.53	98.07	11.0
**DAS**	99.2	10.6	97.7	9.39	105	8.50	98.41	13.5
**OTA**	104	6.92	112	5.70	107	11.50	95.80	10.8
**HT-2**	93.1	20.8	101	10.6	102	14.35	99.23	12.2
**T-2**	94.0	11.0	100	10.8	104	11.10	98.40	13.0
**ENB**	87.0	8.89	89.3	12.1	105	9.54	100.34	13.2
**ENB_1**	100	14.1	95.3	11.5	106	8.95	97.85	11.5
**ENA_1**	106	9.15	106	9.06	107	10.75	96.30	13.7
**ENA**	94.1	14.7	100	12.9	109	12.52	96.72	11.8
**BEA**	100	11.0	96.3	13.7	105	13.42	98.16	12.6
**AOH**	98.6	9.35	97.8	7.56	105	11.58	98.03	13.4
**AME**	95.1	14.7	107	7.67	102	14.73	98.62	12.0
**TEN**	65.9	13.4	97.2	14.3	107	13.29	99.54	13.5
**OTα**	95.7	7.25	102	7.34	104	13.08	98.37	8.62
**HFB_1**	85.4	16.5	99.0	9.69	108	7.58	97.2	7.98
**DH-CIT**	106	10.1	110	15.2	109.15	13.27	102	11.9
**T-2 triol**	83.8	16.5	94.1	11.5	101.04	12.89	99.5	8.44
**FB_2**	107	7.82	112	6.50	109.34	9.77	94.5	11.6
**FB_1**	96.4	14.0	107	12.1	107.90	11.02	95.4	12.6

c) Two different batches of samples – accuracy and precision (n=6)

Analyte	Theoretical Concentration LLOQ		Theoretical Concentration low QC		Theoretical Concentration medium QC		Theoretical Concentration high QC	
	Accuracy [%]	Precision (RSD [%])	Accuracy [%]	Precision (RSD [%])	Accuracy [%]	Precision (RSD [%])	Accuracy [%]	Precision (RSD [%])
**CIT**	111	6.10	108	4.59	104	5.27	91.2	7.97
**α-ZEL**	96.3	14.1	104	7.64	99.2	10.9	100	9.00
**β-ZEL**	89.1	12.8	93.8	12.2	99.6	9.83	100	12.1
**ZEN**	97.0	5.42	101	5.36	104	7.19	98.4	7.61
**β-ZAL**	99.1	10.4	99.6	8.36	102	7.14	99.3	9.20
**α-ZAL**	94.1	12.3	102	5.53	106	6.43	97.6	10.4
**ZAN**	93.0	10.3	106	8.36	102	9.99	98.8	9.07
**NIV**	96.3	10.9	99.9	10.9	106	8.50	97.8	9.57
**DOM-1**	98.3	10.0	97.5	4.92	106	8.48	97.8	7.84
**DON**	98.2	4.04	99.8	3.96	103	6.90	99.0	9.64
**AFB_1**	97.3	13.7	100.0	5.69	104	6.76	98.4	8.36
**AFB_2**	94.9	13.8	99.9	6.41	108	6.72	96.9	10.3
**STC**	96.8	9.99	101	8.13	104	7.01	98.4	6.85
**$AFLM_1$**	90.0	15.0	95.7	17.7	105	8.82	98.3	13.3
**AFG_1**	101	15.7	98.7	12.1	104	10.1	98.7	6.73
**AFG_2**	98.1	6.99	97.9	6.24	106	7.91	97.8	11.2
**15-AcDON**	98.0	7.99	94.9	7.98	105	6.89	98.2	7.53
**3-AcDON**	98.1	10.4	108	13.0	104	7.13	98.2	10.5
**DAS**	100	7.43	98.3	7.12	102	5.90	99.2	7.97
**OTA**	88.6	7.55	104	6.22	105	6.38	97.8	5.61
**HT-2**	97.1	11.3	105	7.31	97.4	7.66	101	5.74
**T-2**	92.0	9.20	102	11.2	102	6.67	99.1	8.27
**ENB**	95.6	13.4	105	9.48	100	9.79	99.8	8.54
**ENB_1**	99.6	6.41	98.3	9.05	103	6.93	98.9	9.36
**ENA_1**	99.8	8.69	97.3	9.51	104	6.74	98.5	9.11
**ENA**	97.2	13.9	99.2	7.94	106	6.83	97.9	7.41
**BEA**	96.8	11.0	99.5	8.22	106	8.17	97.9	11.1
**AOH**	93.9	11.5	103	10.7	105	7.34	97.8	9.28
**AME**	98.9	7.63	101	6.54	100	8.74	99.9	12.4
**TEN**	95.9	9.14	102	2.99	103	5.89	98.6	6.08
**OTα**	89.2	10.9	106	5.47	100	11.9	99.8	7.88
**HFB_1**	95.9	9.62	99.2	8.68	108	6.22	97.2	7.47
**DH-CIT**	87.7	16.9	99.3	11.6	110	12.7	96.2	11.2
**T-2 triol**	89.5	9.98	103	9.05	111	8.43	95.1	8.28
**FB_2**	101	12.5	99.7	8.06	102	8.13	99.3	10.3
**FB_1**	99.4	17.3	99.6	14.5	115	5.57	94.4	10.4

Table S4. Composition of feed, metabolizable energy and the analyses of the selected ingredients of diets, Mix parameters in 1 kg:

Components	%
Wheat bran	4
Post-extraction soybean meal	21
Wheat	10
Rye	15
Corn	35
Barley	11
Corn gluten	1
Premix piglets 10-30	3

Table S5. Analytical composition of the feed: Mix parameters in 1 kg (metabolic energy 13.00 MJ/kg):.

Components	%
Total protein	16
Calcium	0.58
Phosphorus	0.48
Sodium	0.18
Lysine	1.04
Methionine + cystine	0.64
Threonine	0.59
Tryptophan 10-30	0.19
